# Fulminant versus non fulminant acute myocarditis: evolution of functional parameters evaluated with cardiac magnetic resonance

**DOI:** 10.1186/1532-429X-18-S1-P250

**Published:** 2016-01-27

**Authors:** Patrizia Pedrotti, Francesco Moroni, Enrico Ammirati, Angelica Peritore, Angela Milazzo, Giuseppina Quattrocchi, Paolo G Camici, Cristina Giannattasio, Maria Frigerio, Alberto Roghi

**Affiliations:** 1 Health Science Department, Bicocca University, Milano, Italy; 2 grid.416200.1Cardiology and Cardiac Surgery, Niguarda Hospital, Milano, Italy; 3 Cardiothoracic Department, San Raffaele Hospital and Vita Salute University, Milano, Italy

## Background

Acute myocarditis (AM) is an inflammatory disease of the heart muscle. Clinical presentation and outcome are variable, ranging from asymptomatic to fulminant myocarditis (FM), presenting with decompensated heart failure or cardiogenic shock needing inotropic or mechanical circulatory support. Cardiac magnetic resonance (CMR) is increasingly employed in the diagnosis and follow-up (f.u.) of AM. We investigated the differences in functional parameters evaluated with CMR between patients (pts) presenting with FM versus non fulminant AM (NFM), at baseline and f.u.

## Methods

We retrospectively analyzed consecutive pts with AM who underwent CMR (Siemens Avanto, 1.5 T) within 30 days from clinical presentation. Cine, STIR T2-weighted and late-enhancement images were acquired on matching planes. CMR diagnosis of AM was based on Lake Louise Criteria. Indexed LV end-diastolic (LVEDVi) and end-systolic (LVESVi) volumes, LV ejection fraction (LVEF), indexed LV mass (LVmi), indexed RV end-diastolic (RVEDVi) and end-systolic (RVESVi) volumes and RV ejection fraction (RVEF) were calculated for each patient. F.u. scans were similarly analyzed, if available.

## Results

Four pts with FM died and 3 were heart transplanted before performing CMR and thus were excluded from the analysis; none of the pts with NFM died or were transplanted before CMR. In total, 68 pts (age 35 ± 14 years, 10 females) were included: 11 (16%) with FM and 57 (84%) with NFM underwent CMR. Baseline scans were obtained earlier in the NFM group, 5 (3-9) vs 12 (7-21) days after presentation, p = 0.0035. Baseline LVEF was significantly lower in FM compared to NFM, 56% (43-65) vs 64% (59-68),p=0.026. RVEF was lower in FM than in NFM pts, 56% (52-58) vs 63% (58-66),p=0.0024. F.u. scans were available in 47 pts (9 FM, 38 NFM) and were obtained after a median of 169 (110-203) days from baseline scan in FM and 148 (93-242) days in NFM (p=0.78). In FM, LVEDVi was increased at f.u. (from 75 to 77 mL/m^2^, p=0.028) and LVEF improved from 56% to 66% (p=0.028); in NFM LVEDVi and LVEF were unchanged at f.u. (75 vs 75 ml/m^2^,p=0.26 and 64% vs 65%, p = 0.54 respectively). F.u. LVEF and RVEF did not differ significantly between FM and NFM, 66% (60-69) vs 65% (61-69), p=0.79 and 62% (57-68) vs 63% (59-68),p=0.69 respectively. In both FM and NFM LVmi significantly decreased from baseline (from 76 to 66 g/m^2^, p = 0.009, and from 76 to 71 g/m^2^ p < 0.0001, respectively). There was a trend towards a more pronounced reduction of LVmi in FM, but the difference fell short of statistical significance (-17 vs -8 g/m^2^,p=0.13).

## Conclusions

Pts with FM achieved normal LVEF at f.u., despite a lower baseline LVEF, even if a mild LV remodeling was observed at f.u. This might in part be due to a more pronounced decrease in LVmi, likely secondary to more severe myocardial edema in FM, but this observation warrants further investigation and longer f.u. to better understand long term consequences of FM on ventricular remodeling and prognosis.

